# Spatial band-pass filtering aids decoding musical genres from auditory cortex 7T fMRI

**DOI:** 10.12688/f1000research.13689.2

**Published:** 2018-04-04

**Authors:** Ayan Sengupta, Stefan Pollmann, Michael Hanke

**Affiliations:** 1 Sir Peter Mansfield Imaging Centre, School of Physics and Astronomy, University of Nottingham, Nottingham, UK; 2 Department of Experimental Psychology, Institute of Psychology, Otto-von-Guericke University, Magdeburg, Germany; 3 Psychoinformatics Lab, Institute of Psychology, Otto-von-Guericke University, Magdeburg, Germany; 4 Center for Behavioral Brain Sciences, Magdeburg, Germany

**Keywords:** musical-genre decoding, 7 Tesla fMRI, primary auditory cortex, spatial band-pass filtering

## Abstract

Spatial filtering strategies, combined with multivariate decoding analysis of BOLD images, have been used to investigate the nature of the neural signal underlying the discriminability of brain activity patterns evoked by sensory stimulation – primarily in the visual cortex. Previous research indicates that such signals are spatially broadband in nature, and are not primarily comprised of fine-grained activation patterns. However, it is unclear whether this is a general property of the BOLD signal, or whether it is specific to the details of employed analyses and stimuli. Here we applied an analysis strategy from a previous study on decoding visual orientation from V1 to publicly available, high-resolution 7T fMRI on the response BOLD response to musical genres in primary auditory cortex. The results show that the pattern of decoding accuracies with respect to different types and levels of spatial filtering is comparable to that obtained from V1, despite considerable differences in the respective cortical circuitry.

## Introduction

We recently reported ^1^ that spatial band-pass filtering of 7 Tesla BOLD fMRI data boosts accuracy of decoding visual orientations from human V1. We observed this result in comparison to data without any dedicated spatial filtering applied, and spatially low-pass filtered data – a typical preprocessing strategy for BOLD fMRI. This effect was present across a range of tested spatial acquisition resolutions, ranging from 0.8 mm to 2 mm isotropic voxel size (Figure 4 in 1). The bandpass spatial filtering procedure was performed by a difference-of-Gaussians (DoG) filter similar to Supplementary Figure 5 in 1. The frequency bands indicated the presence of orientation-related signal in a wide range of spatial frequencies as indicated by above-chance decoding performance for nearly all tested bands. Maximum decoding performance was observed for a band of 5–8 mm full width at half maximum (FWHM), indicating that low spatial frequency fMRI components also contribute to noise with respect to orientation discrimination.

This finding raises the question whether this reflects a specific property of early visual cortex and the particular stimuli used in 1, or whether it represents a more general aspect of BOLD fMRI data with implications for data preprocessing of decoding analyses. Here, we investigate this question by applying the identical analysis strategy from 1 to a different public 7 Tesla BOLD fMRI dataset ^2^, with the aim of decoding the musical genres of short audio clips from the early auditory cortex.

## Methods

As this study aims to replicate previously reported findings, by employing a previously published analysis strategy on an existing dataset, the full methodological details are not repeated here. Instead the reader is kindly referred to 2, 3 for comprehensive descriptions of the data, and to 1 for details on the analysis strategy and previous findings. Only key information and differences are reported below.

## Stimulus and fMRI data

Data were taken from a published dataset ^2^ which were repeatedly analyzed previously ^4, 5^, and publicly available from the studyforrest.org project of 20 participants passively listening to five natural, stereo, high-quality music stimuli (6 s duration; 44.1 kHz sampling rate) for each of five different musical genres: 1) Ambient, 2) Roots Country 3) Heavy Metal, 4) 50s Rock’n’Roll, and 5) Symphonic, while fMRI data were recorded in a 7 Tesla Siemens scanner (1.4 mm isotropic voxel size, TR=2 s, matrix size 160 *×*160, 36 axial slices, 10% interslice gap). fMRI data were scanner-side corrected for spatial distortions ^6^. Stimulation timing and frequency were roughly comparable to 1: 25 vs. 30 trials per run, 10 s vs. 8 s minimum inter-trial stimulus onset asynchrony in a low event-related design, 8 vs. 10 acquisition runs. Subject 20 was excluded from the analysis due to incomplete data.

## Region of interest (ROI) localization

**Figure 1.**
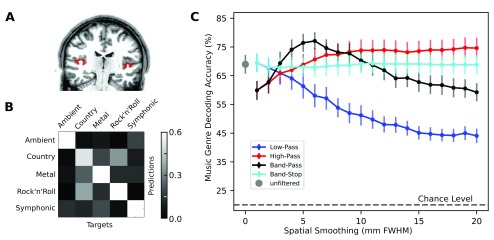
( **A**) Localization of early auditory cortex (transversetemporal gyrus) as shown in coronal slice of a participant (sub-16). ( **B**) Confusion matrix showing the mean performance of the LinearCSVM classifier across all participants in decoding musical genres from early auditory cortex in spatially unfiltered data. ( **C**) Classification accuracy of decoding musical genres across different types and levels of spatial Gaussian filtering. The theoretical chance performance of 20% is shown by the dashed line. *This figure has been generated from original analysis of the dataset made publicly available ^2^ under the terms of the Public Domain Dedication and License.*

Analogous to 1, ROIs were localized separately for each individual brain. ROIs were left and right transversetemporal gyri, as defined by the structural Desikan-Killiany atlas ^7^ from the previously published Freesurfer-based cortex parcellations for all *studyforrest.org* participants ^3^. This ROI approximates the location of primary auditory cortex, including Broadmann areas 41 and 42 ( Figure 1A). The average number of voxels in the ROI across participants was 1412 (std=357).

## fMRI data analysis

Motion-corrected and distortion-corrected BOLD images from the publicly available dataset ^2^ were analyzed. Images for each participant, available from the dataset as the filename pattern of sub*/BOLD/task002_run*/bold_dico_bold7Tp1_to_subjbold7Tp1.nii.gz were already aligned across acquisition runs. Analogous to 1, BOLD images were masked to the defined bilateral ROI, and voxelwise BOLD response were univariately modelled for each run using the GLM implementation in NiPy [v0.3; ^8^] while accounting for serial correlation with an autoregressive term (AR1). The GLM design matrix included hemodynamic response regressors, one for each genre and its corresponding temporal derivatives for improved parameter estimation ^9^, six nuisance regressors for motion (translation and rotation), and polynomial regressors (up to 2nd-order) modeling temporal signal drift as regressors of no-interest. The *β* weights thus computed for each run were *Z*-scored per voxel. Multivariate decoding was performed on these *Z*-scored *β* weights using linear support vector machines [SVM; PyMVPA’s LinearCSVMC implementation of the LIBSVM classification algorithm; ^10, 11^ in a within-subject leave-one-run-out cross-validation of 5-way multi-class classification of musical genres. Leave-one-out cross-validation was performed in order to enable comparison with previous results although it has been recently argued that repeated random splits are a superior validation scheme ^12^. The hyper-parameter *C* of the SVM classifier was scaled to the norm of the data. Decoding was performed using the entire bilateral ROI.

In-line with 1, 13, complete BOLD images were spatially filtered prior to masking and GLM-modeling, as prior results suggest negligible impact of alternative filtering strategies (see Figure S4 in 1). The magnitude of spatial filtering used is expressed in terms of the size of the Gaussian filter kernel(s) described by their FWHM in mm (a conversion of this unit to (cycles/mm) is shown in Supplementary Figure 5 in 1). The image_smooth() function in the nilearn package ^14^ was used to implement all spatial smoothing procedures. The implementations of Gaussian *low-pass* (LP), and *high-pass* (HP) filters, as well as the DoG filters for *bandpass* (BP) and *bandstop* (BS) filtering are identical to those of ^1^ (1 mm FWHM filter size difference).

## Results and conclusions

Figure 1 shows the mean accuracy across 19 participants for classifying the genre of music clips from BOLD response patterns of bilateral early auditory cortex. Compared to visual orientation decoding from V1 ^1^, the mean accuracy of decoding musical genres without dedicated spatial filtering exhibits a substantially higher baseline (for 1.4 mm unfiltered data, mean orientation decoding accuracy was around 35%, whereas mean decoding of musical genres was at around 65%). However, the general pattern of accuracies across all filter sizes and filter types strongly resembles the results of orientation decoding from V1. The superior decoding performance here, in comparison to oriented gabor gratings used for visual decoding, could be the result of the richer naturalistic stimuli with features like pitch, timbre, and speech lead to more separable fMRI activation patterns across genres. LP filtering led to a steady decline of performance with increasing filter size, but does not reach chance level even with a 20 mm smoothing kernel. In contrast to LP filtering, HP filtered data yielded superior decoding results for filter sizes of 4 mm and larger. Congruent with 1, BP filtering led to maximum decoding accuracy in the ≈5–8 mm FWHM band. The accuracy achieved on BP filtered data at 6mm FWHM was significantly higher than that without any dedicated spatial filtering (McNemar test with continuity correction ^15^: *χ* ^2^=33.22, p<10 ^*−*6^). BS filtering led to an approximately constant performance regardless of the base filter size, on the same level as with no dedicated spatial filtering.

In line with Gardumi *et al*. ^16^, these results suggest that BOLD response patterns informative for decoding musical genre from early auditory cortex are spatially distributed and are represented at different spatial scales. However, despite their broadband nature, relevant information seems to be concentrated in the spatial frequency band corresponding to a ≈5–8 mm DoG filter. Most notably, the present findings show a striking similarity to the visual orientation decoding accuracy patterns in V1 ^1^. The origin and spatial scale of signals beneficial for decoding BOLD response patterns are an intensely debated topic in the literature, and various studies have looked at this question in the context of anatomical or topographical structure of visual cortex ^13, 17– 19^. There are substantial differences between the auditory and visual cortex in terms of anatomy, synaptic physiology, and the circuity of cortical layers and their connections with other cortical areas and subcortical nuclei ^20^. The present results indicate that these differences have little impact on the spatial characteristics of those BOLD signal components that are relevant for decoding visual orientation or genre of music. In summary, these findings call for further investigations of neural and physiological signals underlying decoding models that are common across sensory domains, and individual cortical areas. The increasing availability of diverse open brain imaging data can help to aid the evaluation of generality and validity of explanatory models.

## Data and software availability

OpenFMRI.org: High-resolution 7-Tesla fMRI data on the perception of musical genres. Accession number: ds000113b.

Article sources for 7-Tesla fMRI data on the perception of musical genres are available: https://doi.org/10.5281/zenodo.18767 ^21^

“Forrest Gump” data release source code is available: https://doi.org/10.5281/zenodo.18770 ^22^

The codes used in this study for analysis are made openly available: https://doi.org/10.5281/zenodo.1158836 ^23^
