# Supplementary material for: Spatial band-pass filtering aids decoding musical genres from auditory cortex 7T fMRI
Source: F1000Res. 2018 Apr 4;7:142. Originally published 2018 Feb 2. [Version 2] doi: 10.12688/f1000research.13689.2 (PMC5887073; doi:10.12688/f1000research.13689.2)
Supplement: Peer review history

# Peer review: Referee response for version 2

**DOI:** 10.5256/f1000research.15721.r32703

**Published:** 2018-04-04

**Authors:** Cyril R. Pernet

**Affiliations:** 1 Neuroimaging Sciences, Centre for Clinical Brain Sciences, The University of Edinburgh, Edinburgh, UK

As expected, one cannot really address the issue of LOO with 7-8 betas but it's now mentioned and that's all I really wanted to see.

I have read this submission. I believe that I have an appropriate level of expertise to confirm that it is of an acceptable scientific standard.

# Peer review: Referee response for version 1

**DOI:** 10.5256/f1000research.14869.r30542

**Published:** 2018-03-09

**Authors:** David G. Norris

**Affiliations:** 1 Donders Institute for Brain, Cognition and Behaviour, Radboud University Nijmegen, Nijmegen, Netherlands

This article explores the effect of spatial filtering on the decoding accuracy of BOLD fMRI data. The authors conclude that a spatial bandpass filter corresponding to a difference of Gaussians of 6mm improves the decoding accuracy.

 Abstract: 'Reported evidence' is clumsy phrasing how about something like 'previous research'. Use of 'matches' is also ambivalent: do you mean the analysis was similar or the results? Please state the main result and not just that it is similar to that obtained for V1.

 Introduction: The term spatial frequency is used frequently, whereas the difference of Gaussians is described in terms of a FWHM. The units of spatial frequency are 1/mm, so if you are describing band pass filters, cut-offs etc in terms of spatial frequency then please convert to the correct units.

 Stimulus and fMRI data: Please give slice orientation of fMRI acquisition. I believe it was axial, but Figure 1 could mislead people into thinking it was coronal.

 Figure 1 caption: gaussian->Gaussian.

 fMRI data analysis: prior masking -> prior to masking

 Results and Conclusions: These are given with little discussion. Why do you think the decoding accuracy is so much higher for music than for V1. Some discussion of existing literature would also be welcome, also of papers which do not reach the same conclusion as the authors (ref 1).

I have read this submission. I believe that I have an appropriate level of expertise to confirm that it is of an acceptable scientific standard.

## References

[1] GardumiA.IvanovD.HausfeldL.ValenteG.FormisanoE.UludağK.: The effect of spatial resolution on decoding accuracy in fMRI multivariate pattern analysis. *Neuroimage*.2016; 10.1016/j.neuroimage.2016.02.033 32-42 10.1016/j.neuroimage.2016.02.033 26899782

# Response

**Published:** 2018-03-28

**Authors:** Ayan Sengupta

**Affiliations:** Sir Peter Mansfield Imaging Centre, University of Nottingham, UK

**This article explores the effect of spatial filtering on the decoding accuracy of BOLD fMRI data. The authors conclude that a spatial bandpass filter corresponding to a difference of Gaussians of 6mm improves the decoding accuracy.**

**Abstract: 'Reported evidence' is clumsy phrasing how about something like 'previous research'. Use of 'matches' is also ambivalent: do you mean the analysis was similar or the results? Please state the main result and not just that it is similar to that obtained for V1.**

The required phrases have been changed accordingly. 

**Introduction: The term spatial frequency is used frequently, whereas the difference of Gaussians is described in terms of a FWHM. The units of spatial frequency are 1/mm, so if you are describing band pass filters, cut-offs etc in terms of spatial frequency then please convert to the correct units.**

The unit of spatial frequency (FWHM) is kept unaltered in order to maintain parity with other previous publications. But a conversion of units to (cycles/mm) and the implementation of DoG filter is explained in Supplementary Figure 5 of Sengupta et al. 2017. It is now explicitly referred to in the manuscript. 

**Stimulus and fMRI data: Please give slice orientation of fMRI acquisition. I believe it was axial, but Figure 1 could mislead people into thinking it was coronal.**

The words ‘axial slices’ are now mentioned in the description of the acquisition protocol. 

**Figure 1 caption: gaussian->Gaussian.**

The change is incorporated into the manuscript. 

**fMRI data analysis: prior masking -> prior to masking**

This change is done.

**Results and Conclusions: These are given with little discussion. Why do you think the decoding accuracy is so much higher for music than for V1. Some discussion of existing literature would also be welcome, also of papers which do not reach the same conclusion as the authors (ref 1).**

We thank the reviewer for pointing this point in the manuscript. These points are now discussed in the results and conclusion section.

# Peer review: Referee response for version 1

**DOI:** 10.5256/f1000research.14869.r30541

**Published:** 2018-02-08

**Authors:** Cyril R. Pernet

**Affiliations:** 1 Neuroimaging Sciences, Centre for Clinical Brain Sciences, The University of Edinburgh, Edinburgh, UK

This is a good note, replicating previous results from vision to audition - and therefore showing that band-pass is an effective strategy, not specific to visual columns. 

 Method:

- You set a GLM to get beta estimates per genre for each run, and use this (z-scored) for decoding. You mention that you included temporal derivatives, were these ones used in any form? (note you can also correct the hrf amplitude estimates using the temporal derivative https://www.ncbi.nlm.nih.gov/pmc/articles/PMC3896880/)
- You used a within-subject leave-one-run-out cross-validation; as you know I'm sure, Gael showed that LOO tends to be biased (https://arxiv.org/abs/1606.05201). If I understand correctly, that means that the classification is done with 7 betas per class, predict 1 (and rotate). Obviously, that is an issue as K-folds will lead to an even smaller number of beta to use. I don't know if that's addressable here, but worth mentioning the issue (and even better do an alternative sampling scheme if you think it's feasible).

I have read this submission. I believe that I have an appropriate level of expertise to confirm that it is of an acceptable scientific standard, however I have significant reservations, as outlined above.

# Response

**Published:** 2018-03-28

**Authors:** Ayan Sengupta

**Affiliations:** Sir Peter Mansfield Imaging Centre, University of Nottingham, UK

**This is a good note, replicating previous results from vision to audition - and therefore showing that band-pass is an effective strategy, not specific to visual columns.**

**Method:**

**You set a GLM to get beta estimates per genre for each run, and use this (z-scored) for decoding. You mention that you included temporal derivatives, were these ones used in any form? (note you can also correct the hrf amplitude estimates using the temporal derivative https://www.ncbi.nlm.nih.gov/pmc/articles/PMC3896880/)**

The GLM was performed in NiPy [v0.3;8] while accounting for serial correlation with an autoregressive term (AR1) and hrf modelling was set to be ‘canonical with derivative’. The temporal derivatives were included in the model for a better estimation of the beta parameters. This is now explicitly mentioned in the manuscript with corresponding citation.

    

**You used a within-subject leave-one-run-out cross-validation; as you know I'm sure, Gael showed that LOO tends to be biased (https://arxiv.org/abs/1606.05201). If I understand correctly, that means that the classification is done with 7 betas per class, predict 1 (and rotate). Obviously, that is an issue as K-folds will lead to an even smaller number of beta to use. I don't know if that's addressable here, but worth mentioning the issue (and even better do an alternative sampling scheme if you think it's feasible).**

We thank the reviewer for raising this issue with the cross-validation procedure. We agree that the leave-one-out (LOO) cross validation that was performed in this analysis may be biased and repeated random sub-sampling of training set could address the issue. However it has to be noted that the LOO cross validation method was almost exclusively used in all auditory and visual decoding in prior publications. As rightly mentioned by the reviewer, this study is about ‘replicating previous results from vision to audition’ and hence we keep on performing leave-one-out cross validation for making a direct comparison. The caveats of LOO cross validation are now mentioned in the results and conclusion section.
